# Comprehensive pain management strategy for infants with moderate to severe osteogenesis imperfecta in the perinatal period

**DOI:** 10.1002/pne2.12066

**Published:** 2021-12-04

**Authors:** Ricki S. Carroll, Perri Donenfeld, Cristina McGreal, Jeanne M. Franzone, Richard W. Kruse, Catherine Preedy, Joanna Costa, Daniel R. Dirnberger, Michael B. Bober

**Affiliations:** ^1^ Sidney Kimmel Medical College at Thomas Jefferson University Philadelphia Pennsylvania USA; ^2^ Division of Palliative Medicine Department of Pediatrics Nemours Children’s Hospital Delaware Wilmington Delaware USA; ^3^ Division of Orthogenetics Department of Pediatrics Nemours Children’s Hospital Delaware Wilmington Delaware USA; ^4^ Neonatal‐Perinatal Medicine Department of Pediatrics Nemours Children’s Hospital Delaware Wilmington Delaware USA; ^5^ Department of Orthopaedic Surgery Nemours Children’s Hospital Delaware Wilmington Delaware USA

**Keywords:** fracture management, neonatal pain, osteogenesis imperfecta, pain management, pamidronate

## Abstract

Osteogenesis imperfecta (OI) is a rare genetic heterogeneous disorder that causes increased bone fragility and recurrent fractures. For infants with OI and diffuse fractures, pain management, which is nuanced and specific for this population, is of the utmost importance to their neonatal care. Through experience at our center, we have developed a standard approach that has been successful in optimizing survival for these infants during this tenuous period. In this paper, we outline our multidisciplinary approach to pain management for infants with moderate to severe OI during the neonatal period, with emphasis on promotion of fracture healing and adequate pain control.

## 1 INTRODUCTION

Osteogenesis imperfecta (OI), also known as “brittle bone disease,” is a rare heterogeneous genetic disorder characterized by recurrent fractures and increased bone instability and fragility. It has an estimated prevalence of 1 in 15 000‐20 000 births.^1^, ^2^ In 1979, Dr David Sillence developed a classification system for OI that includes four basic types that can be distinguished on the basis of clinical features.^3^ Type I OI, or mild OI, is the most common form of OI and has the mildest presentation with, generally, typical height, no long bone deformities, and the lowest fracture rates. Type II is the most severe form of OI and is classically known as lethal in the perinatal period. Type III is the most severe form of OI in children surviving the neonatal period. Type III OI, or severe OI, is characterized by extreme short stature, growth plate abnormalities, and progressive limb and spine deformities secondary to multiple fractures. Type IV OI, or moderate OI, is an intermediate form between Type I and Type III.^4^ In approximately 90% of patients with OI, an autosomal dominant mutation in one of the genes encoding type 1 collagen (*COL1A1* or *COL1A2*) has been identified.^2^, ^5^ However, with recent advances in understanding the molecular basis of OI, multiple other genetic causes have been identified and the number of subtypes has been expanded to 16.^1^, ^6^, ^7^

The most severe types of OI can often be difficult to distinguish from each other by in utero ultrasonography. This can make it exceedingly difficult to prognosticate survival probability prenatally. In addition, heightened diagnostic awareness and improved treatments, particularly in severe forms, have increased the number of individuals living with OI.^8^ Advancements in technology and the option for life‐sustaining interventions have allowed more infants with “lethal” OI to survive, with no comprehensive guide for how to care for them if or when they do. This points to both the challenge with the historic OI classification system and a shifting prognostic landscape for these infants. From our experience, patient selection is almost impossible prenatally, and medical futility is only applicable when ventilation is not possible, which cannot be fully assessed without a trial of respiratory support with adequate pain management. The infants discussed in this article could ultimately be categorized as having Types II, III, or IV, which we will refer to as moderate to severe OI. Perinatal care for any infant with moderate to severe OI should include conversations with the family to elicit their goals and hopes for their child. This paper will focus on life‐sustaining interventions, although modifications could always be made to align with a family's goals for their infant.

The Osteogenesis Imperfecta team, in conjunction with the neonatology team, at Nemours Children's Hospital in Wilmington, Delaware, has significant experience caring for infants with OI in the moderate to severe spectrum in our neonatal intensive care unit (NICU). Our OI team includes a geneticist, a complex care and palliative care pediatrician, orthopedic surgeons, a genetic counselor, a nurse practitioner, a social worker, and a physical therapist. Our team pairs with the neonatal ICU team to offer the infant comprehensive care. Below, we share our approach to pain management for infants with moderate to severe OI from a multidisciplinary perspective, focused on pharmaceutical, neonatal, and orthopedic needs during this time period. Our success in caring for these infants has highlighted the importance of a standardized clinical method that focuses on adequate pain control and the promotion of fracture healing.

## 2 PHARMACOLOGIC MANAGEMENT

### 2.1 Analgesia

There is no doubt that birth is a traumatic experience for any baby. In infants with OI, this is made even more traumatic by the occurrence of fractures. The mode of delivery does not seem to affect fracture burden, with essentially equivalent numbers of infants sustaining birth fractures through vaginal delivery and cesarean section.^9^, ^10^ However, the likelihood of fractures is affected by the severity of the infant's OI, with at least 90% of newborns with Type III OI and 50% of newborns with Type IV OI experiencing at least one fracture directly related to delivery.^9^, ^10^ Fractures not only cause discomfort but also can have a direct impact on an infant's respiratory status and overall clinical picture.^11^, ^12^, ^13^ Therefore, adequate pain control is imperative to both survival and well‐being of infants with OI.

Assessing an infant's pain level can be challenging; however, there are many validated pain tools established for infants who experience long‐term pain.^14^, ^15^ There is no one scale that is best for infants with OI; what is most important is that one is implemented and that scores are followed to ensure adequate analgesia is reached.^16^, ^17^, ^18^ As‐needed opioids can be used initially following birth, while obtaining images, evaluating fracture burden, and assessing the infant's comfort level. However, an opioid infusion is generally required for adequate pain control during the first few days to weeks of life, depending on the number of and type of fractures present.^16^, ^19^ This is especially important if rib fractures are present, not only for comfort but also to decrease respiratory splinting and promote respiratory efficiency. Additional as‐needed doses of opioids should be used whenever pain scores are elevated or the infant is being moved during this initial period of time.

At approximately 7‐10 days of life, we expect to see woven bone formation at the site of birth fractures develop into a visible callous and stabilize the fracture site. Around this time, infants will typically begin moving their extremities more and showing signs of recovery. Once there is evidence of fracture healing clinically, weaning the opioid infusion can begin, with an ultimate goal of discontinuation. Premedication continues to be beneficial prior to movement that could cause discomfort, including when obtaining images, changing positions, or engaging in physical or occupational therapy. Pain assessment tool scores should continue to be followed to ensure pain is well controlled during this time of healing. Once scheduled opioids have been successfully weaned and discontinued, pain can usually be well controlled with as‐needed acetaminophen. Nonpharmacological measures are also strongly encouraged, including music therapy, environmental modifications, and skin‐to‐skin parental holding. In our experience, long‐term daily opioids are not typically required for these infants. As‐needed opioids are reserved for new significant events, such as a substantial long bone fracture or multiple rib fractures. When opioids are required, dosing is weight based and dependent on the infant's prior exposure and current needs, as is true for any infant.

### 2.2 Pamidronate

Pamidronate is a nitrogen‐containing bisphosphonate that inhibits bone resorption and is widely used to improve bone density and stability in children with moderate to severe OI. Although its mechanisms of antiresorptive action are incompletely understood, pamidronate binds to hydroxyapatite and may directly block its dissolution. In addition, bisphosphonates are internalized into osteoclasts, where they disrupt production of key proteins essential for osteoclast activity. The beneficial effects of these treatments initiated in infancy are known to include fracture reduction^20^; improved growth in infancy^20^, ^21^; improvements in vertebral shape, size, and bone mineral density; and improved gross motor function.^22^ Furthermore, Antoniazzi et al^20^ and Plotkin et al^23^ reported the elimination of signs of bone pain in infants and children, such as crying with handling, within a week of starting bisphosphonate therapy. In the immediate newborn period, once acute fracture pain has dissipated, management of more generalized bone pain is important; therefore, we advocate starting the first pamidronate cycle as soon as is practical.

At our center, we have adopted the protocol reported by Glorieux et al,^24^ namely pamidronate diluted in normal saline (0.1 mg:1 mL NS at maximum concentration), infused over 4 hours. In the first 2 years of life, we administer 0.5 mg/kg/d for 3 consecutive days, cycled every 8 weeks. In severely affected infants in the NICU who have umbilical venous access, we aim to give the first pamidronate course prior to umbilical catheter removal (roughly 7‐10 days), to reduce the risk of early fractures from peripheral line placement. The primary adverse effect of pamidronate is a febrile acute phase reaction that can be seen during the first cycle and possibly mediated by tumor necrosis factor‐alpha (TNF‐α).^23^ To minimize this risk, as described by Munns et al,^25^ on the first day of the first infusion, we decrease the dose to 25% (0.125 mg/kg), and, on days 2 and 3 of this first infusion, we decrease the dose to 50% (0.25 mg/kg). We also give premedication with acetaminophen before each dose during the first cycle.

Due to the inhibition of calcium release from bone, hypocalcemia is a known potential side effect of pamidronate therapy and requires close monitoring during and following bisphosphonate therapy. For this reason, it is important that age‐appropriate calcium intake is established before proceeding with treatment. We typically delay the first infusion until an infant is receiving stable enteral or parenteral nutrition with adequate calcium intake. During this first cycle, we follow ionized calcium prior to each daily dose of pamidronate. Despite these precautions, hypocalcemia can occur but is often transient.^26^ In our experience, hypocalcemia may be more severe among infants born prematurely. It may be necessary for enteral or intravenous calcium supplementation to be given during pamidronate therapy for up to 72 hours after infusion. We monitor ionized calcium in all infants following their first cycle until levels begin to rise approximately 72 hours following the final infusion. If supplementation is necessary, we monitor calcium until it is stable in the normal range for age.

Adverse respiratory events have also been reported, associated with the first cycle of pamidronate. In a report by Munns et al,^25^ 4 of 59 (7%) infants with severe OI and preexisting respiratory compromise developed respiratory distress during the initial pamidronate cycle. These respiratory issues were well managed with bronchodilators. The subsequent cycles of pamidronate did not exacerbate the infants' respiratory status. Accordingly, we defer cycle initiation in infants with acute respiratory instability.

## 3 NUTRITIONAL CONSIDERATIONS

Optimal enteral nutrition should be achieved and maintained as early as possible, to support bone strength and growth and provide the necessary substrates for fracture healing. Nutritional goals include the provision of calcium and phosphorus at optimal ratios parenterally until early supplementation is possible as well as the early initiation and advancement of enteral feedings. We target lower volume and calorie delivery for patients with OI and other skeletal dysplasias to avoid excessive adiposity. These infants have decreased activity and caloric demand and have limited skeletal growth compared with age‐matched controls. Caloric delivery equivalent to unaffected infants will result in excessive fat deposition, abdominal competition, and excessive fluid retention, all of which may compromise respiratory efforts and lead to higher respiratory support requirements. In addition, avoiding fluid overload will help mitigate the use of loop diuretics, which contributes to excessive calcium and phosphorus losses. We have found that achieving an average weight accrual of 5‐10 g/d is an appropriate growth target for infants with the most severe forms of OI. This can typically be achieved by maintaining a target of approximately 100‐110 mL/kg/d, providing 75‐80 kcal/kg/d.

## 4 RESPIRATORY AND HEMODYNAMIC MONITORING

Many of these patients will require respiratory support for survival of the neonatal period, including endotracheal intubation and mechanical ventilation.^13^, ^27^ The care team should anticipate this requirement and be proactive about intubation early in the course of respiratory failure. Every effort should be made to ensure first‐pass success when it comes to intubation; this includes adequate sedation and empiric neuromuscular blockade. Video laryngoscopy (VL) is recommended to mitigate neck extension and optimize visualization, if those resources and experienced personnel are available. The infant with OI is not the patient on whom to attempt VL for the first time. If VL is not an option, the infant with OI can be successfully and safely intubated by careful standard direct laryngoscopy technique. A fear of cervical fracture is no reason not to provide the necessary respiratory support, if appropriate precautions are taken.

In the early newborn period, when laboratory sampling is needed more often, and when hemodynamic instability is most likely, maintaining umbilical venous and arterial access is recommended immediately following birth. This allows for invasive continuous hemodynamic monitoring rather than frequent handling for vital sign evaluation. Furthermore, heel‐stick and peripheral blood sampling increases the risk of fractures from extremity manipulation, heel massage, and excessive manual pressure. For the same reason, maintaining central venous access is preferred over repeated peripheral IV placement.

Once the infant is stable, invasive hemodynamic monitoring can be discontinued. There is great concern around the use of blood pressure cuffs in patients with OI, which is understandable as case reports have described fracture events.^28^, ^29^, ^30^ However, in a study by Sullivan et al^31^ of 37 patients, including 12 with severe OI and 7 with moderate OI, who underwent 98 procedures, there were no iatrogenic fractures resulting from blood pressure cuff or tourniquet use. Given this, while at our hospital we try our best to avoid the use of both of these interventions when not necessary, if at any point either of these tools is required for continued optimal care of the infant, we will not hesitate to use either of these techniques. Similarly, we try our best to avoid blood sampling and to have stable, long‐term intravenous access whenever possible; however, when peripheral access or blood sampling is required, careful attention to avoiding joint hyperextension, hyperflexion, and manual pressure is important. Having a low threshold for the use of preprocedure sedation and analgesia will improve procedural success, avoid infant agitation, and minimize the risk of fractures.

## 5 FRACTURES

### 5.1 Fracture mitigation

Fracture mitigation should pervade all aspects of care, beginning at birth. A sign is placed above the infant's bed to indicate to others the fracture risk with handling. Newborns are placed on an egg crate mattress in the delivery room and remain on one throughout the hospital course. While having the parents hold their baby is strongly encouraged, we recommend the infant be moved on, and remain on, an egg crate mattress or pillow in the parents' arms, especially during this first week of life. This allows the infant to be moved en bloc and minimizes axial or torsional stresses on the extremities or trunk, thereby minimizing pain and facilitating healing. When lifting or moving the infant off of the egg crate, great care is taken to use large surface areas and wide‐open hands instead of pincer grasps, to decrease chance of fractures.^13^, ^19^ Other fracture mitigation considerations in daily care include the use of loose‐fitting clothing, such as sleep sacks, and avoiding sleeves that require arm manipulation and twisting to don and remove; strict adherence to clustering hands‐on care, including position changes and diaper changes; and avoiding unnecessary movement, such as daily weights.^16^, ^19^ We generally weigh these infants two or three times per week in the early course, and as little as once per week as the infant becomes more stable and minor weight changes are less likely to affect management.

### 5.2 Fracture management

Despite careful handling and optimized medical care, fractures may still occur. The management of these fractures is a fundamental feature of neonatal OI care and pain management. An audible or palpable snap of the bone may be heard or felt in some instances, but not for the majority of fractures in this age group. Additional signs of a fracture include sudden onset of discomfort, unexplained fussiness, lack of movement of an extremity, swelling, and ecchymosis.

Assessment of a neonatal fracture includes a clinical examination of the area of concern, including an evaluation for the above noted signs of a fracture. Fracture assessment also includes an examination of the soft tissue envelope, as it would be quite unusual to encounter threatening of the skin with a fracture in an infant with moderate to severe OI. The examination is focused on evaluation for newly apparent or worsened deformity, presence of instability or crepitus at the fracture site, evaluation of blood flow to the distal aspect of the affected extremity, and movement of the fingers or toes distal to the suspected fracture site. We recommend avoiding reflexively obtaining radiographs of the area of interest. Such radiographs incur radiation exposure and discomfort associated with positioning, and they do not alter the treatment for the suspected fracture, provided the above assessment has demonstrated a closed injury with a reassuring neonatal neurovascular examination.

**FIGURE 1:**
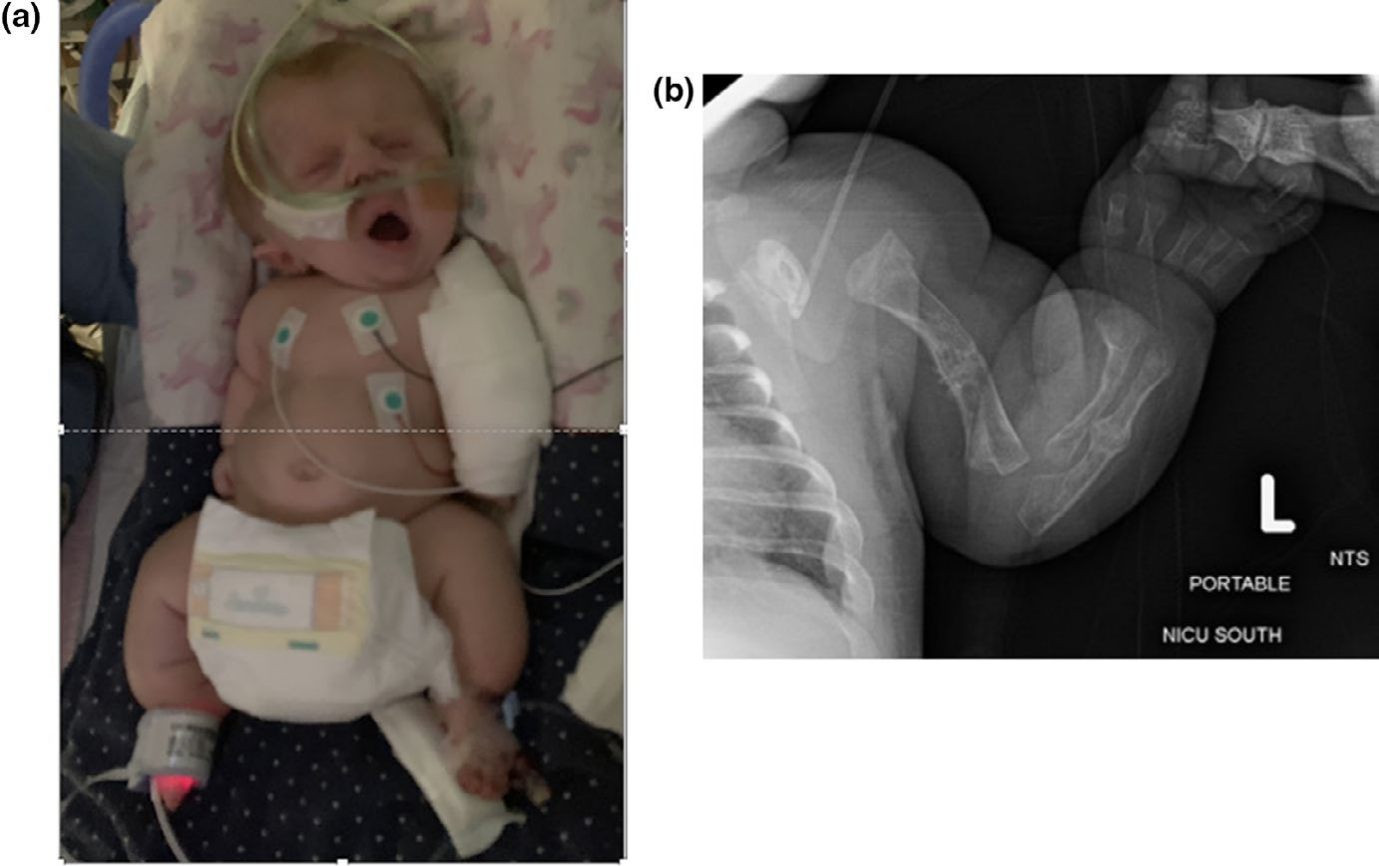
a, 4‐month‐old girl with a cotton wrap on the left upper extremity for a presumed left upper extremity fracture. b, Left upper extremity radiograph from 8 days of age; radiographs of the left upper extremity were not pursued with fracture at 4 months of age; wrap was removed approximately 10 days following the fracture with resumption of baseline comfort level and movement and use of the left upper extremity. Photograph used with parental consent

**FIGURE 2:**
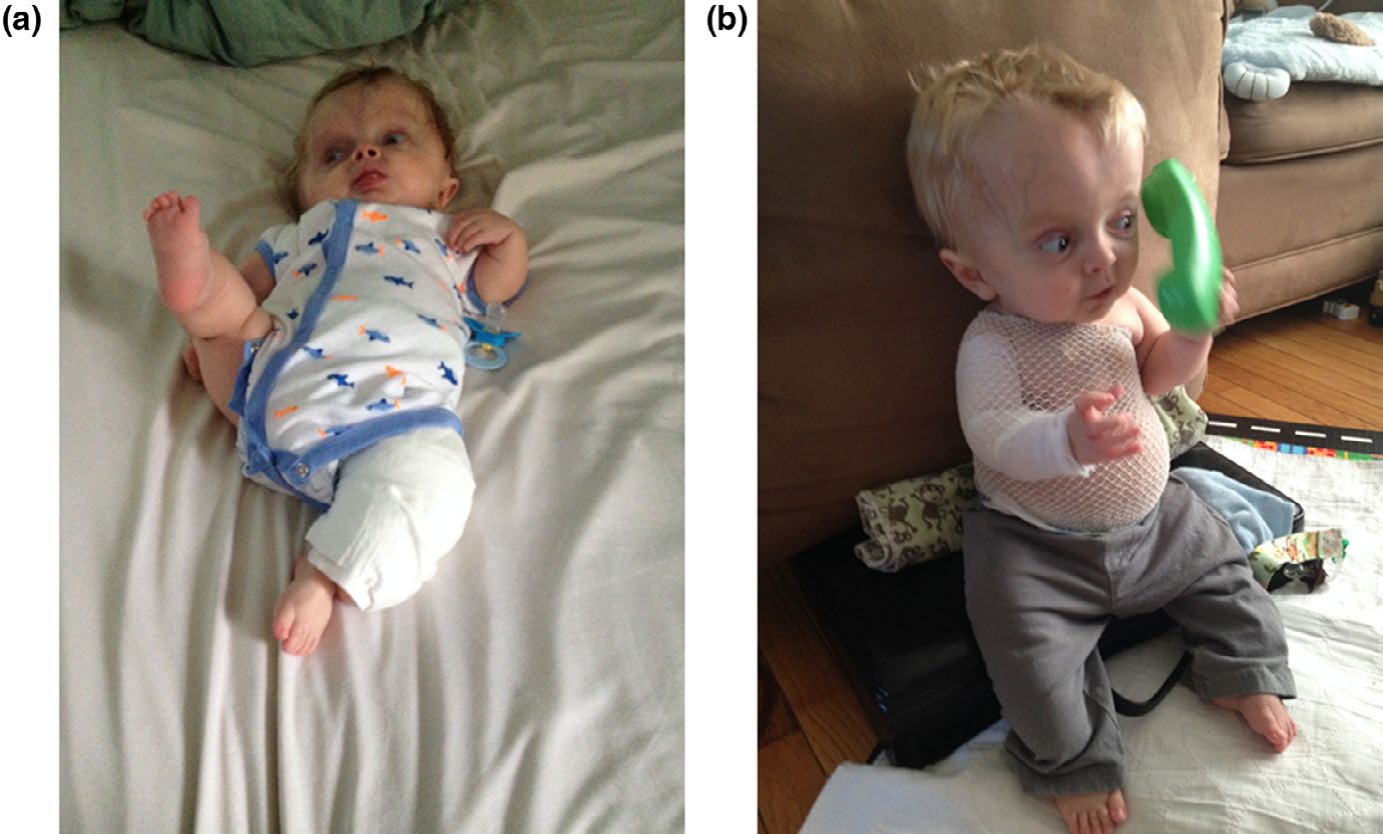
Patient with severe osteogenesis imperfecta as a neonate with a left lower extremity cotton wrap (a) and once discharged home from the neonatal intensive care unit with a right upper extremity cotton wrap and a swathe (b). Photograph used with parental consent

**FIGURE 3:**
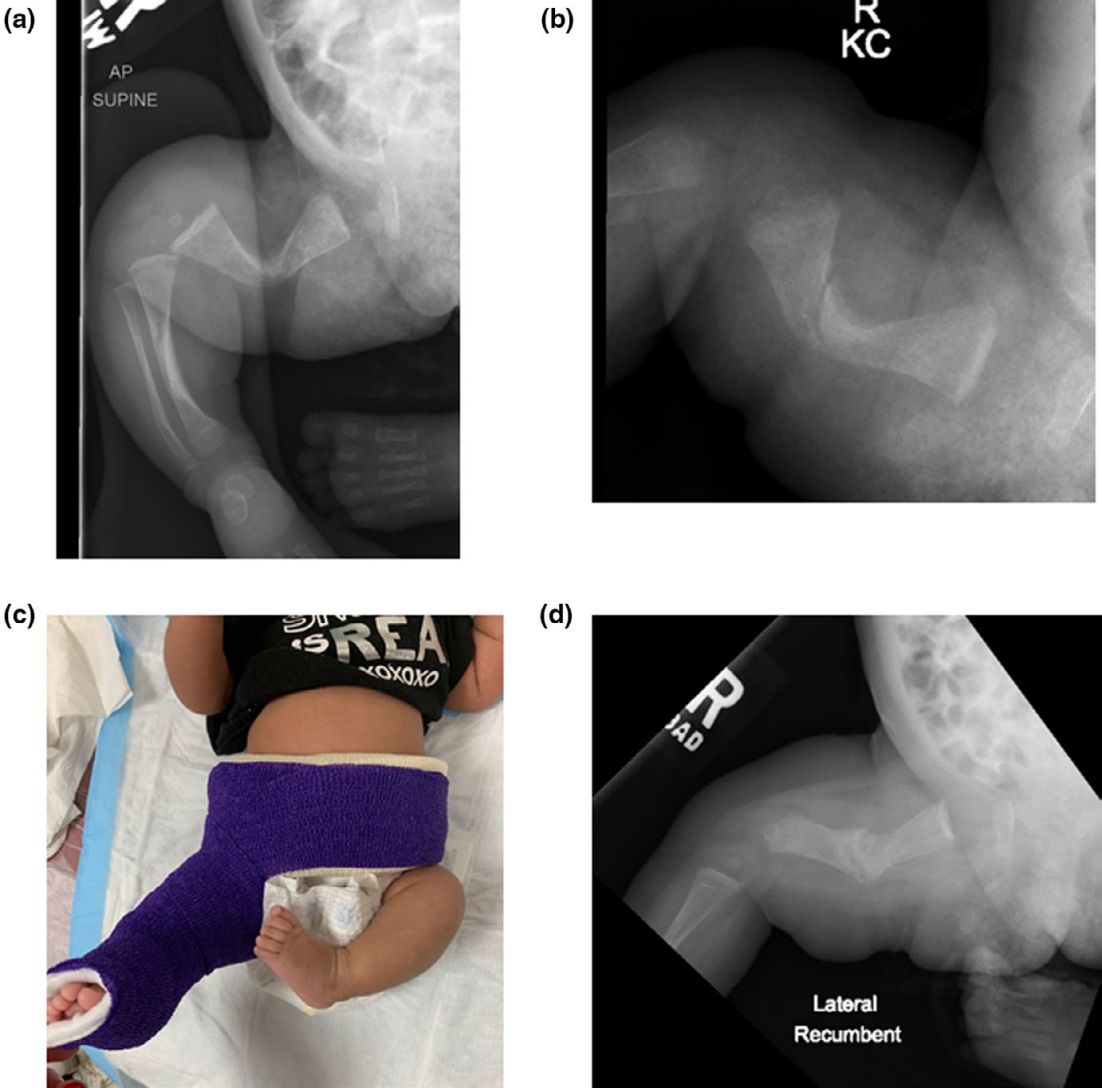
a & b, Two views of the right femur in a 20‐day‐old girl with moderate osteogenesis imperfecta and a right femur fracture. c, Soft wrap immobilization with overlying flexible cohesive bandage and a foam wrap around the lower trunk to complete a soft spica wrap. d, Right femur radiograph 4 weeks later when soft spica wrap was removed

For fractures that occur beyond the first 7‐10 days of life, the initial line of treatment to improve comfort is positioning of the affected extremity on a pillow or soft protective surface and avoiding movement of the extremity. Additional immobilization may be achieved with a lightweight soft wrap for the extremity. In our experience, a wrap of multiple layers of cotton undercast padding provides adequate stability in a neonate and is lightweight (Figures 1 and 2). We prefer to use the cotton material (not synthetic), as it will tear if undue tension is applied. As the infant grow, additional stability may be achieved with an overlying wrap of a flexible cohesive bandage (such as CoFlex^®^, Andover Healthcare, Salisbury, MA, USA) or the addition of a lightweight splint (Figure 3). It is important that an experienced member of the care team or family member with proper education place a potentially compressive wrap, such as flexible cohesive bandage, to avoid it being applied too tightly and jeopardizing circulation to the affected extremity. Following immobilization, capillary refill of the digits must be examined and routinely checked. In addition to providing immobilization and comfort, these types of lightweight soft wraps serve as a reminder of the fracture to the care team and family and may be removed when discomfort with transfers has abated and spontaneous movement of the affected extremity has resumed. There is little to no role for heavy plaster or fiberglass casts for fracture management in the neonate with OI. Although infants with moderate to severe deformity with bowing and recurrent fractures of the extremities may require realignment and intramedullary rodding of the long bones during the early childhood years, surgical intervention does not play a role in the management of the vast majority of fractures in the neonatal period.

## 6 FAMILY SUPPORT

Having an infant with OI can be accompanied by a wide range of emotions. Partnering with the parents and allowing them to be an integral part of the care team is essential. With such a significant, life‐altering diagnosis, the importance of proper and early positive bonding cannot be understated. Allowing the parents to hold their infant at the bedside is one of the most effective ways to promote and develop bonding. Use of an egg crate mattress or pillow allows for safe transfer from bed to lap and back early in the infant's course, and we encourage this as much as possible. We involve our supportive services from early on, including social work and spiritual services. When a family is grappling with medical decisions around life‐sustaining technology or needing an additional layer of support, we will involve our palliative care team.

The care of an infant with OI involves a multidisciplinary and specialized team. Education and support are offered through each discipline. Education for the parents on how to handle their infant is often provided by the bedside nurses, who have had extensive experience with this patient population. Physical therapists are available to assist with positioning for holding. If an infant can take feeds by mouth and a mother is interested in breast feeding, a lactation consultant will pair with a physical therapist to ensure safe and comfortable positioning for nursing. Closer to discharge, our orthopedic team provides education around splinting and fracture care at home.

Finally, for many families, we find that having an OI community is immensely helpful. With permission, we often try to connect families who have been through similar circumstances in hopes that they can be a support to one another. We also encourage families to connect with groups on social media and through the Osteogenesis Imperfecta Foundation.

## 7 CONCLUSION

Infants with severe OI have unique and specific care needs in the perinatal period that require a multidisciplinary team approach. Published guidance on optimal management during the first few weeks of life for infants with OI has been limited to date. In this paper, we outline the care plan and pain management considerations for an infant with moderate to severe OI in the first few weeks of life. Future literature is needed focusing on care beyond this period of time.

## CONFLICT OF INTEREST

Drs. Bober and Carroll are investigators for Biomarin, Pfizer, QED, and Ascendis. Dr Bober also serves as a consultant for these companies. Drs. Franzone and Kruse are consultants for Orthopediatrics. For Jeanne M. Franzone: This work was supported by an Institutional Development Award (IDeA) from the National Institute of General Medical Sciences of the National Institutes of Health, Bethesda, MD [grant number: U54‐GM104941] (PI: Binder‐Macleod). No competing financial interests exist for the rest of the authors on this paper.
